# IL-17 in Rheumatoid Arthritis and Precision Medicine: From Synovitis Expression to Circulating Bioactive Levels

**DOI:** 10.3389/fmed.2018.00364

**Published:** 2019-01-14

**Authors:** Marie Robert, Pierre Miossec

**Affiliations:** Department of Clinical Immunology and Rheumatology, Immunogenomics and Inflammation Research Unit EA 4130, University of Lyon 1, Hôpital Edouard Herriot, Lyon, France

**Keywords:** synovitis, rheumatoid arthritis, interleukin-17, interleukin-17 inhibition, precision medicine

## Abstract

Interleukin (IL)-17A has a direct contribution in early induction and late chronic stages of various inflammatory diseases. *In vitro* and *in vivo* experiments have first characterized its local effects on different cell types and then its systemic effects. For instance, IL-17 axis is now identified as a key driver of psoriasis through its effects on keratinocytes. Similar observations apply for rheumatoid arthritis (RA) where IL-17A triggers changes in the synovium that lead to synovitis and maintain local inflammation. These results have prompted the development of biologics to target this cytokine. However, while convincing studies are reported on the efficacy of IL-17 inhibitors in psoriasis, there are conflicting results in RA. Patient heterogeneity but also the involvement of mediators that regulate IL-17 function may explain these results. Therefore, new tools and concepts are required to identify patients that could benefit from these IL-17 targeted therapies in RA and the development of predictive biomarkers of response has started with the emergence of various bioassays. Current strategies are also focusing on synovial biopsies that may be used to stratify patients. From local to systemic levels, new approaches are developing and move the field of RA management into the era of precision medicine.

## Introduction

Interleukin (IL)-17A is a pro-inflammatory cytokine that contributes to the pathogenesis of several auto-immune and inflammatory diseases (1). *In vitro* and *in vivo* experiments have identified IL-17 effects on various cell types explaining its involvement in early induction and late chronic stages of many diseases. For instance, IL-17A acts on keratinocytes to induce the expression of several chemokines leading to the recruitment of immune cells that characterized psoriasis (2). Furthermore, in rheumatoid arthritis (RA), the most prevalent chronic inflammatory disease (3), IL-17A acts locally on synoviocytes and osteoblasts contributing to synovitis and joint destruction (4, 5).

These observations have prompted the development of biologics targeting IL-17A and various strategies are currently being tested (2). In psoriasis, inhibitors of IL-17A axis bring a clear benefit in patient care management. Among diseases affecting joints, IL-17 inhibitors are effective in active ankylosing spondylitis and psoriatic arthritis, whereas conflicting results are reported for RA with a high degree of heterogeneity in response (6–9). To potentiate the use of such therapies in RA, an effort is needed to precisely identify patients that would respond to IL-17A inhibition. Current strategies are focusing on the development of biomarkers (5, 10) but also on synovial biopsies (11) to explain patient heterogeneity and treatment response.

The present review discusses the effects of IL-17A on synovium, its regulation and current strategies to detect bioactive IL-17A. Regarding the role of IL-17 in RA pathogenesis, these observations emphasize that this cytokine and its inhibitors should now be considered in the development of precision medicine in RA.

## IL-17 and Synovitis

### The IL-17 Family

#### IL-17A, IL-17F, and IL-17E

The IL-17 family is composed of six members: IL-17A to IL-17F. The IL-17A was the first isoform discovered in 1993. Initially described as cytotoxic T lymphocyte-associated antigen 8, a product of T cells in rodents, the effects of human IL-17A were then characterized (12, 13). One of its earliest documented biological activities was its effects on RA synoviocytes (14). Then, it was shown that this cytokine promotes granulopoiesis and protects the host against bacterial and fungal infections (1).

Among the IL-17 family, IL-17A and IL-17F share the greatest homology with a 50% sequence identity and can be secreted as homodimer or heterodimer (15, 16). Many of the effects of IL-17A and IL-17F are found similar even if IL-17F is usually less active at inducing inflammation (1).

**Figure 1:**
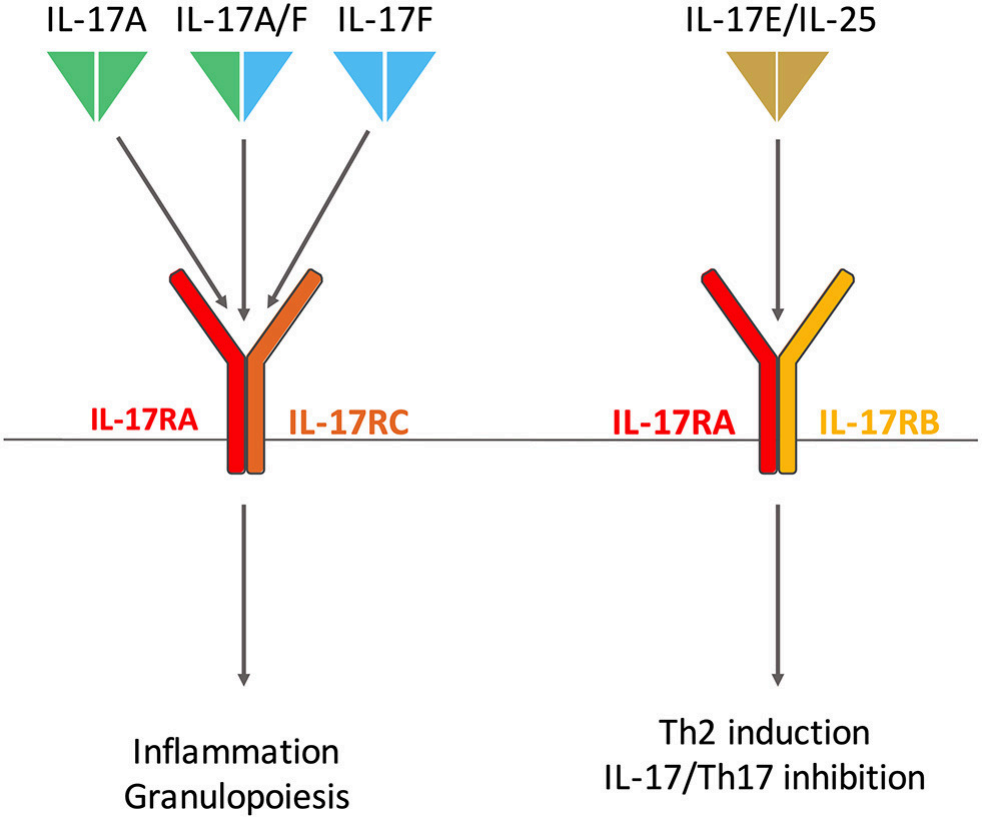
Interleukin (IL)-17 and Receptor family involved in rheumatoid arthritis. IL-17A and IL-17F homodimers and the IL-17A/IL-17F heterodimer bind the same receptor composed of IL-17RA and IL-17RC subunits. IL-17RA is also involved in IL-17E (also known as IL-25) receptor with IL-17RB. IL-17A/F and IL-17E have distinct biological effects, the first triggers inflammation and granulopoiesis; the latter promotes T-helper (Th) 2 responses in host defense against parasites and allergy. IL-17E also regulates Th17 inflammatory response.

Conversely, IL-17E (also known as IL-25) has the lowest homology with IL-17A with only 20% sequence identity (17). IL-17E is a mediator of T-helper (Th) 2 cell responses especially in host defense against parasites (18) and allergy (19). In addition, it also regulates Th17 inflammatory response and IL-17 function (20) (Figure 1).

#### IL-17 Producing Cells

The first cellular source of IL-17 was identified in 1999 as a particular subtype of CD4+ T cells (21). Th17 cells were finally described in 2005 in the mouse being different from the classical Th1 and Th2 cells (22). The differentiation of Th17 cells is a multi-step process involving transforming growth factor ß, IL-21, IL-1ß, IL-6, and IL-23 in humans (23, 24). The lineage-specific transcription factor retinoic acid receptor-related orphan receptor (RORc, RORγt in mice) is required for the differentiation (23). Other subsets of immune cells can produce IL-17 including γδ T cells, natural killer cells, invariant natural killer T cells, innate lymphoid cells and CD8+ T cells (2).

#### IL-17 Receptor Family and Signaling

The first receptor of IL-17 to be identified was discovered in 1995 (25). The IL-17 receptor (IL-17R) family now includes 5 subunits, from IL-17RA to IL-17RE (26). IL-17A, IL-17F, and IL-17A/F bind the same receptor composed of IL-17RA and IL-17RC subunits (27). IL-25 binds a receptor made of IL-17RA and IL-17RB (28). Despite their opposite biological effects, IL-17A and IL-25 share a common receptor chain, an important point to consider when targeting IL-17RA in clinic (2) (Figure 1).

Upon ligand binding, the association of IL-17R with Act1 (also known as connection to iκB kinase and stress-activated protein kinases) induces the recruitment and the ubiquitination of tumor necrosis factor (TNF)-receptor associated factor-6 triggering nuclear factor-κ B (NF-κB), CCAAT/enhancer binding protein-ß, CCAAT/enhancer binding protein-δ and mitogen-activated protein kinase pathways. IL-17R and Act1 also activate extracellular signal-regulated kinase-5. These two signaling pathways mediated by IL-17 induce the transcription of inflammatory genes. IL-17 signaling also increases mRNA stability of IL-17 target genes (29). mRNA stabilization is one of the process by which IL-17 and other cytokines synergize, as described below for TNFα (30). Interestingly, peptide that blocks the interaction between Act1 and IL-17RA decreases both IL-17A and IL-25-induced inflammation (31).

### Production by and Effects of IL-17 on Synovitis

Many changes occur in the RA synovium, which is characterized by hyperplasia, neoangiogenesis and local infiltration by immune cells (32, 33). These modifications trigger the destruction of cartilage and bone. The role of IL-17 in the synovitis pathogenesis was first characterized by observations on RA explants. Then, its effects on synovial and bone biopsies and *in vitro* are described.

#### IL-17 and Th17 Cells in RA Synovial Tissue Pathobiology

Shortly after the description of IL-17, observations on synovial tissues and fluids of RA patients suggest that this cytokine may be involved in joint destruction. Indeed, immunostaining of the synovial tissues of RA patients demonstrates that a subset of CD4+CD45RO+ memory T cells produces IL-17; these IL-17 positive cells being not detected in synovial tissue from osteoarthritis (OA) patients. Moreover, concentration of IL-17 in synovial fluid is also higher in RA patients than in OA, trauma and gout patients (34). Interestingly, there is a spontaneous secretion of IL-17 by RA synovium compared with OA and normal synovium (35, 36). IL-17 synovial membrane mRNA level predicts damage progression (37). Double-immunofluorescence studies show that RORc co-localized with IL-17A and IL-17F staining suggesting that Th17 cells participate to the local cytokine production. IL-17A and IL-17F producing-cells are detected in the lymphocytic infiltrates and in hyperplasic lining cells of RA synovium (30). The recruitment of Th17 cells to the joint leads to interactions with local cells that perpetuate chronic inflammation (38). Specifically, cell interactions between Th17 cells and synoviocytes are crucial as they lead to a massive production of IL-17. The interaction molecule podoplanin contributes widely to this high IL-17 secretion (39, 40). *In vitro* and *in vivo* experiments show that IL-17A and IL-17F-producing cells have a plasma-cell like morphology (30, 41). This morphology has been associated with increased secretion *in vitro* and probably *in vivo*. Experiments on synovial explants from RA show that the Th2 cytokines IL-4 and IL-13 completely inhibit the production of IL-17 (35).

All together, these findings suggest a local production of IL-17 in RA synovium, mainly mediated by Th17 cells. The interactions between local mesenchymal cells and Th17 cells are crucial for a higher and more sustained production.

#### Effects of IL-17 in RA Pathogenesis

Having characterized the production of IL-17 in RA synovitis and the cells involved, IL-17 effects on synovial and bone explants are now described.

Structural damage in RA includes cartilage destruction and bone erosion (42). Cartilage damage is partially induced by synovial cytokines such as IL-17. Experiments on RA synovial samples show that IL-17 triggers the production of IL-6, leukemia inhibitory factor (LIF) and macrophage inflammatory protein (MIP)-3α/chemokine (C-C motif) ligand-20 by RA synovium (35, 43, 44). Moreover, the addition of an anti-IL-17 antibody to RA synovium cultures significantly decreases matrix metalloproteinase (MMP)-1 production, collagenase activity but not tissue inhibitor of MMP (TIMP)-1 production suggesting the direct contribution of IL-17 to joint destruction (45). The MMP/TIMP system plays a role in the collagen tissue turnover; a shift toward MMP production suggests degradation of the collagen framework. MMP-1 induces collagen degradation and the release of carboxy-terminal telopeptides. IL-17 increases carboxy-terminal telopeptides production in RA synovium explants, an effect that is reversed when adding an anti-IL-17 antibody (45, 46). Keeping with this, the C-pro-peptide of type I collagen, representing the production of type I collagen as part of repair efforts, is inhibited when adding IL-17 to RA synovium (46). All together, these results suggest that IL-17 promotes cartilage destruction at the expense of cartilage synthesis.

As mentioned above, RA also leads to bone erosion and particularly to early juxta-articular bone loss (42). Keeping with the results on RA synovium, IL-17 alone, and more in combination with IL-1 or TNFα, increases the production of IL-6 by RA bone explants (46, 47). In addition, IL-17 reduces bone formation and increases its destruction (46).

**Figure 2:**
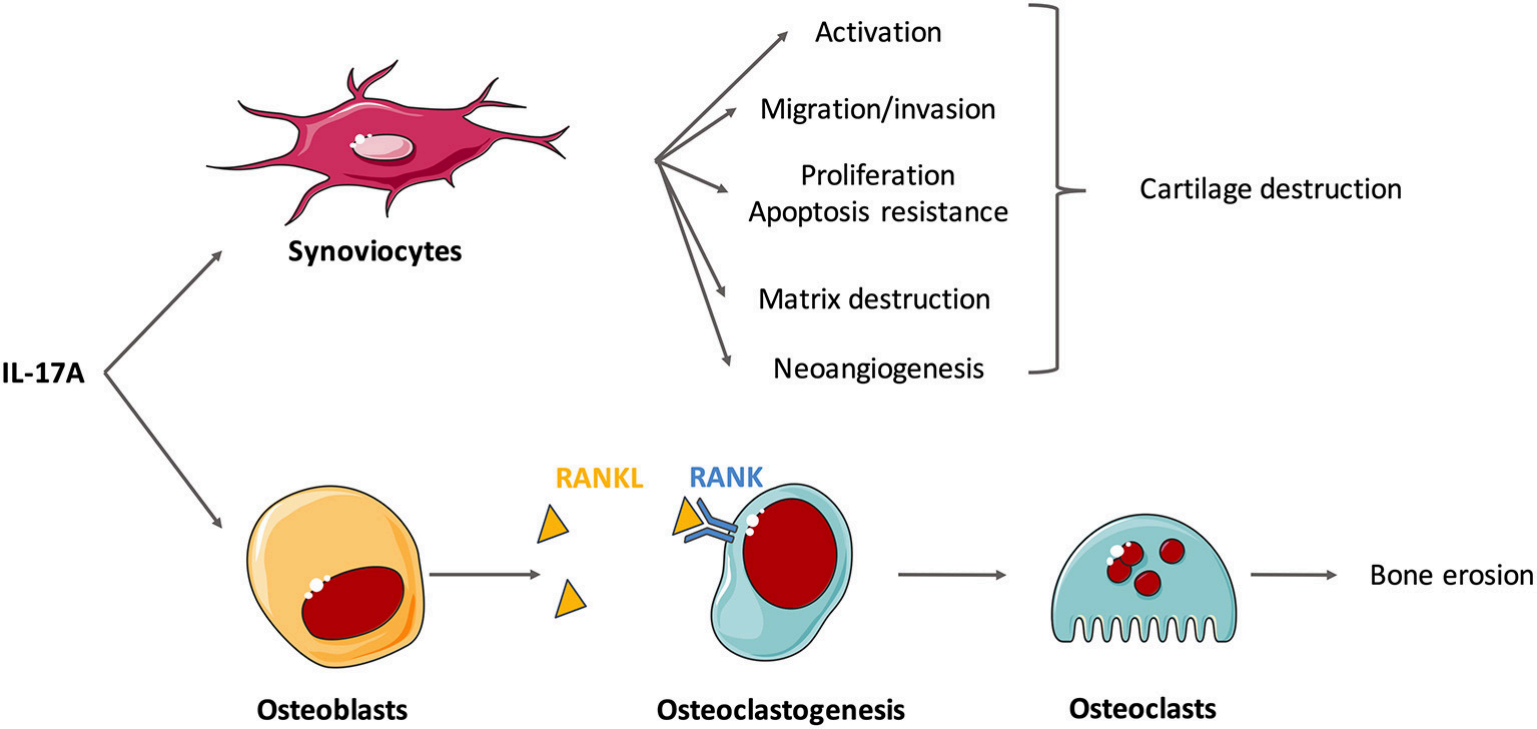
Effects of interleukin (IL)-17A on isolated cells involved in the synovitis. Rheumatoid synovium is characterized by hyperplasia, neoangiogenesis and excessive inflammation. IL-17A mediates cartilage and bone destruction that occurs in rheumatoid arthritis (RA) mainly through its action on synoviocytes and osteoblasts. *In vitro* experiments show that IL-17A induces synoviocyte activation (e.g., production of IL-6 and IL-8), migration and invasion that promote cartilage destruction. Through the induction of matrix metalloproteinases (MMP), IL-17A induces matrix destruction. It also favors proliferation and apoptosis resistance and the neoangiogenesis required for pannus development. In addition, IL-17 promotes receptor activator of NF-κB ligand (RANKL) expression on osteoblasts that binds to RANK, activates osteoclastogenesis and finally triggers bone erosion by osteoclasts.

To go further into the comprehension of IL-17-induced destruction, effects of IL-17 on isolated cells are now described (Figure 2). IL-17A and IL-17F induce synoviocyte activation with increased cytokine and chemokine production, especially of IL-6 and IL-8 (4, 48–51). Moreover, IL-17 triggers synoviocyte migration and promotes an invasive phenotype that favors tissue destruction (33, 52, 53). Tissue destruction includes cartilage matrix destruction and bone erosion. Matrix destruction is mainly mediated by MMP. Among them, MMP-1,−2,−9, and−13 are induced by IL-17 in RA synoviocytes and chondrocytes (45, 54).

Bone remodeling roughly depends on the balance between the activity of osteoclasts, that favor destruction, and osteoblasts, that promote bone formation. IL-17 promotes the expression of receptor activator of NF-κB ligand (RANKL) on osteoblasts and synoviocytes and then activates RANK signaling in osteoclasts (1, 55, 56). These results suggest that IL-17 plays a role in osteoclastogenesis, thereby promoting bone destruction (34, 57). Moreover, IL-17A could inhibit osteoblast and osteocyte activity *in vitro* but this should be confirmed (58).

Neoangiogenesis is crucial for pannus development in RA synovium. IL-17 is involved in this process inducing the production of vascular endothelial growth factor by synovial fibroblasts (59, 60). The RA synovium is also characterized by hyperplasia of synovial lining cells. IL-17 stimulates synoviocyte proliferation (61). This excessive proliferation combined with apoptosis resistance causes synovial hypertrophy. More specifically, IL-17 up-regulates anti-apoptotic genes and down-regulates pro-apoptotic genes (61, 62). IL-17 alone, and especially when combined with TNFα, increases the expression of the anti-apoptotic adhesion molecule Amigo 2 (63) and that of synoviolin, that prolong the survival of RA synoviocytes (50, 64). IL-17 also impairs apoptosis through activation of autophagy (65).

Therefore, observations on synovial and bone samples from RA patients and *in vitro* experiments confirm the role of IL-17 in synovitis.

## Regulation of IL-17 Function

**Figure 3:**
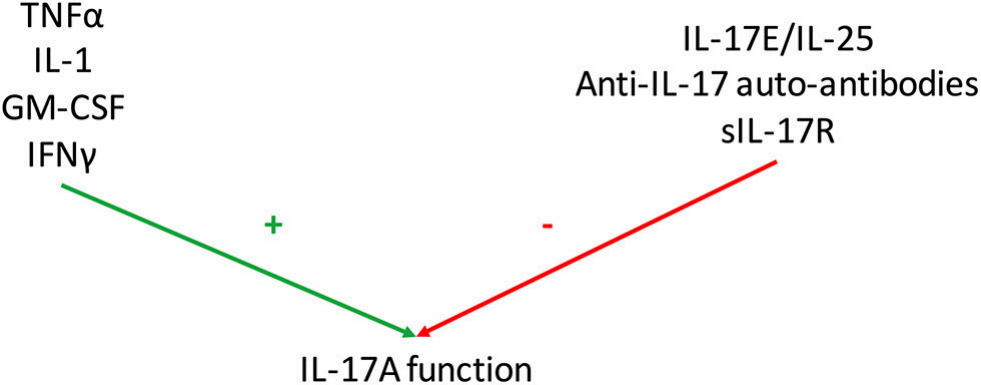
Regulation of Interleukin (IL)-17 function. Various mediators regulate IL-17A function. Some have additive or synergistic effects with IL-17A as tumor necrosis factor-α (TNFα), IL-1, granulocyte-macrophage colony stimulating factor (GM-CSF) and interferon (IFN) γ. Conversely, IL-25 (also known as IL-17E), anti-IL-17 autoantibodies and soluble IL-17 receptor (sIL-17R) inhibit IL-17 function.

Even if IL-17 effects on RA synovitis are clear, some mediators interfere with this system by regulating positively and negatively IL-17 function (Figure 3).

### Synergistic Effects

#### IL-17 and TNFα

Concomitant with the description of human IL-17A (13), experiments showed that TNFα potentiates the effect of IL-17A on IL-6 and IL-8-induced secretion by rheumatoid synoviocytes (14, 56). Similar results were obtained on RA synovium explants (37, 44). Moreover, IL-17F also synergizes with TNFα (4, 48). Mechanisms underlying this synergistic interaction were later described when IL-17A and IL-17F were shown to induce TNF receptor II expression and production. Microarrays analysis reveal that almost 90% of genes modified by the combination of IL-17A and TNFα showed a pattern of additivity and 1% of synergy (4, 30). Interestingly, IL-17 and TNFα mainly synergize through the induction of mRNA stabilization independently of TNF-receptor associated factor-6 (66, 67). Some genes synergistically induced by this combination are of importance in RA (e.g., IL-6, IL-8, chemokine (C-C motif) ligand-20, etc …). For instance, IL-17 and TNFα promote an invasive phenotype in synoviocytes (53) but also neutrophil survival (68).

#### IL-17 and IL-1

IL-1 is involved in RA pathogenesis through bone and cartilage destruction (69). *In vitro* experiments on RA synoviocytes show a synergistic effect of IL-17 and IL-1ß on the production of IL-6 whereas an additive effect is observed for LIF production (43, 70). Interestingly, IL-17 and IL-1ß induce synergistically chemokine (C-C motif) ligand-20 production, which in turn recruits Th17 cells (44). The synergistic effect of IL-17 and IL-1ß is also demonstrated in RA bone explants where the two cytokines increase bone destruction and reduce its formation (46). Similar results are observed with the collagen-induced arthritis mouse models (71, 72). Blocking of both IL-1ß and IL-17A with a bi-specific antibody appears to reduce joint inflammation, destruction and synovial proliferation notably through the reduction of NF-κB activation (72). While IL-1 inhibitors (anti-IL-1ß antibody or soluble type I IL-1 receptor) have modest effect in RA, it would be of interest to develop biological agents that block both IL-17 and IL-1 (69).

#### IL-17 and GM-CSF

Granulocyte-macrophage colony stimulating factor (GM-CSF) is produced by many cell types (e.g., myeloid cells, tissue-resident cells) and plays a key role in the differentiation of myeloid cells and in the production of neutrophils, eosinophils and monocytes. GM-CSF is also involved in adaptive immunity. *In vivo* experiments show that GM-CSF is involved in RA pathogenesis (69). GM-CSF level is also increased in synovial fluid and blood from RA patients (73). In experimental arthritis, the combination of IL-17 and GM-CSF shows complementary and local additive effects and induces a more severe phenotype (74).

#### IL-17 and IFNγ

Interferon (IFN) γ plays a role in anti-infectious host defense, in inflammatory and in auto-immune diseases (75). IL-17A and IFNγ have an additive effect on IL-6 secretion by RA synoviocytes (14). Experiments on other cell types show that the combination of IL-17 and IFNγ increases the production of IL-6, IL-8, intracellular adhesion molecule-1 and nitric oxide (50, 76).

### Antagonist Effects

#### IL-17 and IL-25

As described above, IL-17A and IL-25 bear the lowest homology and their receptors share the common chain IL-17RA (1).

In a mouse model of type I diabetes, IL-25 effect is similar to that of anti-IL-17 to reduce peri-islet CD4 and CD8 T-cell infiltrates while increasing the proportion of the Treg cell population. Interestingly, only IL-25 treatment reduces the amount of autoreactive Th2 and Th17 cells in delayed recurrent autoimmunity. This study highlights the potential shift induced by IL-25 into the Th17/Treg balance (77).

Administration of IL-25 reduces collagen-induced arthritis development in mice and suppresses Th17 cell responses in an IL-13 dependent manner (78). Similar observations are made in experimental autoimmune encephalomyelitis mice where IL-13 is also required to induce Th17 suppression (20).

IL-25 level is higher in serum and synovial fluid from RA patients compared with OA patients and healthy controls (78, 79). Similarly, IL-25 level is correlated with disease activity and with inflammatory cytokines (e.g., TNFα, IL-1ß, IL-17A, IL-6) in RA patients. Moreover, when stimulated peripheral blood mononuclear cells from RA patients are treated with recombinant IL-25, Th17 cells and IL-17A expression are inhibited and that of IL-4 increased (78).

Interestingly, there is a spontaneous secretion of IL-25 by RA synoviocytes that is delayed compared with the production of IL-6. Similar results were obtained in a model known to mimic the inflammatory site of RA synovium (synoviocytes/peripheral blood mononuclear cells coculture), IL-25 production being delayed compared with that of IL-17A. In turn, IL-25 can inhibit IL-17A function acting as a receptor antagonist (79).

Considering the interaction between IL-17A and TNFα, IL-25 reduces the production of IL-6 induced by these two cytokines. Interestingly, IL-17A and TNFα decrease IL-25 production while TNFα alone increases IL-17RB in synoviocytes, being a potential way for TNFα to regulate inflammation (79). Indeed, IL-17RB is required for IL-25 signaling that in turn controls Th1 and Th17 responses and inhibits monocyte-derived inflammatory cytokines (20, 79–81).

All of these results suggest that IL-25 acts as a regulatory pathway in response to inflammation to then down-regulates excessive Th17 and IL-17 immune response.

#### IL-17 and Autoantibodies Against IL-17

Autoantibodies against pro-inflammatory cytokines were first described for IL-1α and constitute a marker of good prognosis in RA (82, 83). They bind their antigen and form immune complexes with the cytokine. Anti-IL-17 antibodies are absent in healthy controls while there are detected in almost 40% of RA patients. As opposed to bioactive IL-17A, anti-IL-17 antibodies are increased in non-severe RA and so linked to a better prognosis. As expected, higher titers of immune complexes are detected in non-destructive compared with destructive RA (10).

#### IL-17 and sIL-17R

The expression of cytokines is regulated by various mechanisms. For instance, IL-1 receptor antagonist binds to IL-1 receptors, competitively antagonizes the binding of IL-1 and finally decreases its biological effects (84). Soluble type II IL-1 receptor also acts as an inhibitor of IL-1 function (85).

Although not fully demonstrated, it makes sense to consider the contribution of soluble IL-17R (sIL-17R) in the regulation of IL-17 function. Interestingly, sIL-17RB is increased in alveolar echinococcosis infected patients compared with controls and its level is correlated with disease severity. Conversely, sIL-17RA shows an opposite trend. These variations of soluble receptors may silence the IL-25 mediated response, thereby promoting disease progression (86).

This example, far from the RA, illustrates that sIL-17R is involved in the modulation of IL-17 levels. *In vitro* and *ex vivo* experiments with RA samples have shown that the combined inhibition of IL-17, IL-1 and TNFα with soluble receptors increased the degree of response (44, 87, 88).

Therefore, many mediators regulate positively or negatively IL-17 function; these results are summarized in Figure 3. Considering all these interactions, it remains a challenge to detect the specific effect of IL-17 both at local and systemic level.

## IL-17 Detection

### From Local Production to Circulating Levels

It makes sense that patients with high level of IL-17 would be more sensitive to an anti-IL-17 inhibitor. This concept was developed after the emergence of TNFα inhibitors. In a majority of patients, this treatment leads to the reduction of symptoms, inflammation and bone destruction. However, around 30 % of these RA patients do not respond. To better understand this observation, a bioassay was developed to evaluate TNFα bioactivity before treatment (89). It is based on the ability of synoviocytes to produce IL-6 in response to TNFα (90). Indeed, 60% of patients have a good ability of their plasma to induce IL-6 production before infliximab therapy (a TNFα inhibitor), this production being inhibited 4 h after the first infliximab infusion. Another pattern of patients has moderate or no IL-6 production before infusion, therefore no inhibition by infliximab. The difference of IL-6 production before and 4 h after the first infliximab infusion is correlated with clinical response. This may explain the heterogeneity in treatment response to TNFα inhibitors (89). Interestingly, intra-articular administration of etanercept (a TNFα inhibitor) results in a significant improvement of the composite change index compared to placebo in RA and psoriatic arthritis patients. Serum etanercept levels were comparable between composite change index good and non-responders, thus indicating that local inhibition of TNFα would be effective (91).

Similar experiments would be of interest for IL-17 since IL-17 systemic inhibitors show heterogeneous results in RA. Interestingly, IL-17A synovial fluid levels are higher than serum levels in early RA cases, suggesting that local production may be reflected by circulating levels (92).

More recently, studies from the Pathobiology of Early Arthritis Cohort have been set up with the aim to define from synovial biopsies and blood samples the involvement of cellular/molecular signatures in determining clinical phenotypes (11, 93, 94). For instance, in early RA, synovial transcripts correlating with disease activity (disease activity score-28/C-reactive protein) are significantly enriched in TNFα-induced genes and predict poor response to first-line therapy (95). Considering the important interpatient heterogeneity, such approaches on synovial biopsies may be used to stratify patient for tailored drug delivery strategies (94), especially in the case of IL-17 inhibitors where results showed a high heterogeneity. Indeed, IL-17 and its receptor are up-regulated within synovial ectopic lymphoid structures and further contribute to the chronicity of local inflammation (64, 96). These structures have the ability to function as germinal centers and there is a significant association between their presence and erosive disease. Aggressive treatments are recommended for these patients to prevent the onset of erosions. Considering the key role of IL-17 in the formation of these ectopic lymphoid structures, IL-17 inhibition would be of interest in these patients selected with a synovial biopsy (97, 98).

### Methods to Detect IL-17 (ELISA and Bioassay)

Considering the results described above, there is no doubt that IL-17 is involved in RA pathogenesis. However, IL-17A circulating levels measured by ELISA vary a lot across studies, from undetectable to pg/ml or even ng/ml concentration (99, 100). Moreover, these tests do not detect the bioactive form that is crucial since there are circulating inhibitors (IL-25, anti-IL-17 autoantibodies, sIL-17R) and activators of IL-17 (TNFα, IL-1, GM-CSF, IFNγ) (Figure 3). To measure the level of bioactive IL-17A, a cell-based bioassay was developed on the ability of RA synoviocytes to produce IL-6. RA synoviocytes are exposed to plasma samples and IL-6 production is measured with or without an anti-IL-17 antibody (35). The test was then extended to human endothelial cells that are able to produce IL-8 in presence of IL-17A (5). By blocking IL-17A, it allows to quantify its specific contribution in the production of pro-inflammatory cytokines.

## Toward Precision Medicine in RA

While many studies are performed to identify predictive biomarkers of RA development (e.g., cigarette smoking, infection), another issue is also to predict which therapy is the best suited for patients that have developed RA (101).

### Predictive Biomarkers of Response to IL-17 Inhibition

Using the bioassay described above, bioactive IL-17A is higher in RA patients compared with healthy controls and its level is correlated with destruction (5). As mentioned earlier, anti-IL-17 antibodies and immune complexes are elevated in non-destructive RA (10). Detection of these biomarkers represents an interesting tool to identify patients with an IL-17 driven disease that could respond better to IL-17 inhibitors.

### Identification of Patients That Would Benefit From Anti-IL-17

In RA patients, a meta-analysis shows the superiority of secukinumab (anti-IL-17A) and ixekizumab (anti-IL-17A) compared with placebo based on American College of Rheumatology (ACR)-20 and ACR50 clinical response. However, it does not reach statistical significance for ACR70 response and analysis of individual response rate shows a high degree of heterogeneity. Moreover, brodalumab (anti-IL-17RA) is not effective in achieving ACR20 (2, 102). These observations rely on different explanations. First, immunohistochemical analysis reveal a high variability of IL-17A, IL-17F and their receptor expression in RA synovitis (103). IL-17 inhibition would not be sufficient in these patients with low expression of IL-17. Then, different strategies are developed to block the IL-17 pathway with anti-IL-17A, anti-IL-17A/F and anti-IL-17RA (2). These antibodies may encounter some pitfalls; for instance, the inhibition of IL-17RA could inhibit the anti-inflammatory effect mediated by IL-25 (28). The dual inhibition with bi-specific antibodies against TNFα and IL-17A would have been of interest to prevent their synergistic interaction but recent papers show no clear benefit, especially when compared to TNFα inhibition alone (104–106). The structure of the dual inhibitor and the respective location of the two binding sites have to be considered.

Therefore, as for TNF inhibitors, an effort is needed to identify RA patients that would benefit from IL-17 targeted therapies. The development of predictive biomarkers of response to IL-17 inhibitors is beginning; for instance, the cell-based bioassay detecting bioactive IL-17A is of interest but only constitutes the spearhead of more research. IL-17 expression in synovial tissue may be another way to stratify patients to potentiate the beneficial effect of these inhibitors (11, 96, 103). Even if robust evidence is still needed to confirm the use of such biomarkers in clinical routine, these strategies can move the field of RA management into the new era of precision medicine in the future.

## Conclusion

IL-17A is involved in early induction and late chronic stages of various inflammatory diseases. The inhibition of its signaling brings a clear improvement in psoriasis, psoriatic arthritis and in ankylosing spondylitis treatment but results are less convincing in RA. However, *ex vivo* and *in vitro* studies clearly show that IL-17A is one of the culprit that perpetuates local inflammation in synovium and especially in RA. Explanations of such unexpected results may come from the many mediators that modulate IL-17 function, with either agonist or antagonist effects. The significant heterogeneity of IL-17 expression between patients also imposes a stratification of them to identify the ones that could benefit from IL-17 inhibitors. The development of predictive biomarkers as bioactive IL-17 or anti-IL-17-autoantibodies or the use of synovial biopsies still requires robust evidence but would be interesting to turn the page to precision medicine in RA.

## Disclosure

PM holds a patent on the IL-17 bioassay.

## Author Contributions

MR: writing and figures. PM: concept and proof reading.

### Conflict of Interest Statement

The authors declare that the research was conducted in the absence of any commercial or financial relationships that could be construed as a potential conflict of interest.

## Abbreviations

IL: interleukin
RA: rheumatoid arthritis
Th: T-helper
ROR: retinoic acid receptor-related orphan receptor
IL-17R: IL-17 receptor
TNF: tumor necrosis factor
NF-κB: nuclear factor-κ B
OA: osteoarthritis
LIF: leukemia inhibitory factor
MIP: macrophage inflammatory protein
MMP: matrix metalloproteinase
TIMP: tissue inhibitor of MMP
RANKL: receptor activator of NF-κB ligand
GM-CSF: granulocyte-macrophage colony stimulating factor
IFN: interferon
sIL-17R: soluble IL-17R
ACR: American College of Rheumatology.
